# Flexural Behavior of Polyurethane Concrete Reinforced by Carbon Fiber Grid

**DOI:** 10.3390/ma14185421

**Published:** 2021-09-19

**Authors:** Hongjian Ding, Quansheng Sun, Yanqi Wang, Dongzhe Jia, Chunwei Li, Ce Ji, Yuping Feng

**Affiliations:** 1 College of Civil Engineering, Northeast Forestry University, Harbin 150040, China; dinghj@nefu.edu.cn (H.D.); 2019011066@nefu.edu.cn (Y.W.); 724106875@nefu.edu.cn (D.J.); lichunwei@nefu.edu.cn (C.L.); 2 Material Science and Engineering College, Northeast Forestry University, Harbin 150040, China; 2019210350@nefu.edu.cn

**Keywords:** carbon fiber grid, flexural bearing capacity, four-point bending test, polyurethane concrete

## Abstract

In view of the problems of traditional repair materials for anchorage concrete of expansion joints, such as ease of damage and long maintenance cycles, the design of polyurethane concrete was optimized in this article, which could be used for rapid repair of concrete in anchorage zone of expansion joints. A new type of carbon fiber grid–polyurethane concrete system was designed, which makes the carbon fiber grid have an excellent synergistic effect with the quick-hardening and high-strength polyurethane concrete, and improved the flexural bearing capacity of the polyurethane concrete. Through the four-point bending test, the influence of the parameters such as the number of grid layers, grid width, and grid density on the flexural bearing capacity of polyurethane concrete beams was tested. The optimum preparation process parameters of carbon fiber grid were obtained to improve the flexural performance of polyurethane concrete. Compared with the Normal specimen, C-80-1’s average flexural strength increased by 47.7%, the failure strain along the beam height increased by 431.1%, and the failure strain at the bottom of the beam increased by 68.9%. The best width of the carbon fiber grid was 80 mm, and the best number of reinforcement layers was one layer. The test results show that the carbon fiber grid could improve the flexural bearing capacity of polyurethane concrete. The carbon fiber grid–polyurethane concrete system provides a new idea for rapid repair of the anchorage zone of bridge expansion joints, and solves the problems such as ease of damage and long maintenance cycles of traditional repair materials, which can be widely used in the future.

## 1. Introduction

The bridge expansion joint’s anchoring zone is the most prone to damaged device in the bridge structure, and it is difficult to rapidly repair without interrupt traffic. Polyurethane concrete is a composite material composed of two components with polyurethane as the binder and cement and aggregate as the filler [1,2], which has the characteristics of quick-hardening, high initial strength, and excellent comprehensive mechanical strength, but it has high usage costs and construction process requirements. The repair method can be applied for rapid repair of concrete in anchorage zone of bridge expansion joint, which solves the problems such as ease of damage, poor driving comfort, long maintenance cycles [3,4].

Currently, many studies are more focused on the research of polyurethane cement, and aggregates have not yet been added to polyurethane concrete composite materials. Kexin Zhang evaluated the bonding performance of polyurethane cement by steel bar pullout test and flexural strength tests of concrete axial tension [5]. Jifeng Zhang et al. showed the fitting result of the relationship between compressive strength, flexural strength, and density of polyurethane cement is a quadratic equation [6]. Nan Yang mixed chopped carbon fiber into polyurethane cement (PUC) and delayed the crack development of carbon fiber polyurethane cement (CPUC) composites, which effectively improved the split tensile strength and flexural strength of CPUC. He also used silica fume instead of some cement, which formed a good CPUC gradation, and improved the density and compressive strength. Furthermore, they analyzed the influence of ash concentration ratio, carbon fiber content, and silica fume content on compressive strength, splitting tensile strength, and flexural strength [7]. The T-beams were reinforced by wire mesh–polyurethane cement (WM–PUC) composite material, and tested through the four-point bending test. The reinforced beams showed significantly higher yield load, ultimate load, and stiffness, and because of excellent bonding performance of PUC, no other adhesive are needed in the reinforced beam [8,9].

In this study, the carbon fiber fabric grid was designed to improve the flexural performance of polyurethane concrete composites, so as to play an important role in future maintenance and reinforcement fields. The fiber fabric grid is a unidirectional or bidirectional, natural or synthetic fiber tow arranged in a regular matrix with high strength and minimum weight. So it can be used as a reinforcement method for existing reinforced concrete components, and is suitable for the situations where steel bars cannot be used because of potential corrosion problems [10]. Meier, Bakis et al., and Zaman et al. proposed the potential for reinforcement with fiber-reinforced polymer (FRP) and discussed its uses in different reinforcement applications [11,12,13]. Pendhari and Bank studied how reinforcement of FRP acts on different structural elements [14,15]. The evaluation of the effectiveness of reinforced materials is helpful to the research of better reinforced materials and technology. Al-Jelawy and Mackie compared the CFRP-PU system with the CFRP-EP system through single lap shear tests of FRP bonded to concrete, small-scale concrete beams (without reinforcement of steel and FRP), small-scale FRP-strengthened beams (without reinforcement of steel), and large-scale RC girders strengthened with FRP. The results show that the polyurethane composite material makes the FRP sheet have a better stress distribution along the interface between FRP and composite material, which improves the performance of the composite material system [16]. Fiber reinforced cement-based composites (FRCM) have been used as an additional reinforcement technology, and the test results proved strengthening existing reinforced concrete elements with FRCM is technically feasible [17]. Especially, the FRCM effect is obvious, which leads to the increase of strength and the decrease of ductility [18,19]. Furthermore, the selection criteria of reinforcement materials depend on certain factors, such as the durability of materials, the convenience of field operation, and cost-effectiveness. In order to develop better reinforcement materials, it is necessary to understand the properties of different materials.

Carbon fiber has many advantages such as excellent durability, high tensile strength and strong corrosion resistance. There is no requirement regarding the thickness of the protective layer of the corresponding concrete elements as long as they meet the transfer bond stress. Therefore, the carbon fiber fabric grid has obvious advantages in structural repairs [20,21]. In addition, the bonding between the carbon fiber grid and polyurethane concrete is realized by the cohesiveness of polyurethane and the mechanical bite force formed by the aggregates of polyurethane concrete passing through the gap of the grid. The combination of carbon fiber grid and polyurethane concrete forms an organic–inorganic composite material, which has better performance than the cement-based composite material, and solves the problem that steel bars in concrete are prone to corrode.

At present, there is relatively little research on the synergy of polyurethane concrete and carbon fiber grid, and the influence of carbon fiber grid on the flexural performance of polyurethane concrete has not been studied by predecessors. In this paper, the influence of the carbon fiber grid on the flexural performance of polyurethane concrete is discussed, which has a certain reference value for the future research and implementation of the carbon fiber grid–polyurethane concrete system.

## 2. Materials and Methods

### 2.1. Preparation of Experiment Materials

#### 2.1.1. Test Substrate and Reinforced Materials

Polyurethane is generally defined as the polymer compound containing repeated polyurethane bond structure units -[-NH-CO-O-]- on the polymer backbone [22]. The polyurethane structure is -[-CO-NH-R-CO-O-R-O-]n-, usually formed by the polymerization of a binary or polyisocyanate with two or more active hydroxide compounds through a stepwise polymerization.

The organic polyurethane prepared by the reaction of combined polyether and PAPI is used as the main body. The more common and inexpensive cement is used as the filler, which is filled in the polyurethane and mixed with the aggregate to form an organic–inorganic composite material with high strength and toughness.

**Figure 1:**
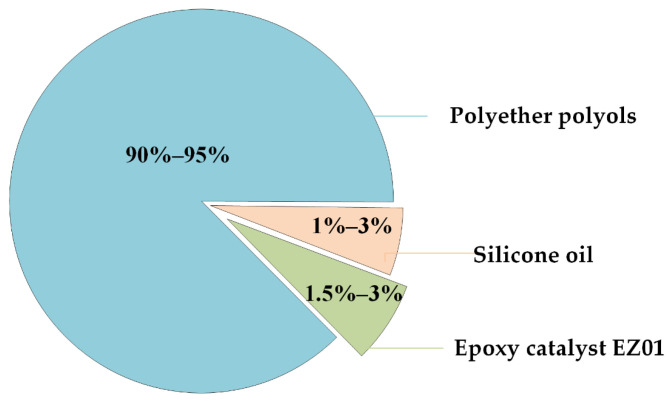
Material composition of combined polyether.

**Figure 2:**
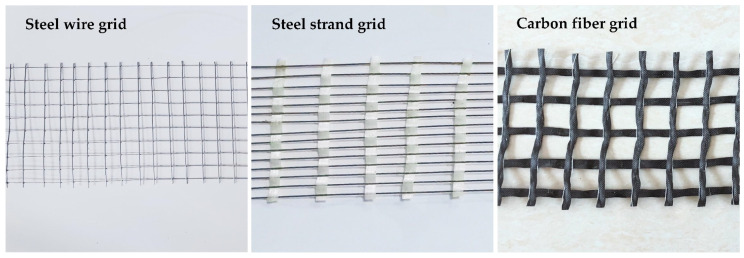
Reinforced materials.

(1) PAPI: Polymethylene polyphenyl polyisocyanate (industrial grade), macromolecule, increased structural integrity, and flexibility.
(2) Combined polyether: Produced by Shandong Yisheng Polyurethane Co., Ltd. (Zibo, China), the main ingredients are polyether polyol, silicone oil, epoxy catalyst EZ01, and a colorless transparent liquid at room temperature. The main material composition of the combined polyethers is shown in Figure 1, while the physical and chemical properties are shown in Table 1.
(3) Aggregate gradation: AC-10 fine-grained concrete typical gradation [23] and the specific particle size and ratio are shown in Table 2.
(4) Cement: Portland cement (average particle size = 1.455 µm), produced by Yatai Group Harbin Cement Co, Ltd. (Harbin, China). According to the T0502-2005 cement fineness test method, T0505-2020 cement setting time test method, and T0506-2005 cement mortar strength test method (ISO method) in “Testing Methods of Cement and Concrete for Highway Engineering” (JTG 3420-2020) [24]. Under the condition of 20 ± 2 °C in the laboratory, the physical indicators of cement were tested. Three samples were taken for each test indicator; the final result is the arithmetic average of the three samples. The physical and mechanical properties of cement are shown in Table 3.
(5) Reinforced material: There are three kinds of reinforced materials in this test: carbon fiber grid, steel-strand grid, and steel-wire grid. The carbon fiber grid is woven from multiple 24 K carbon fiber bundles. In the 24 K fiber type, each fiber bundle includes 24,000 carbon fiber precursors. The carbon fiber precursors used in this experiment were polyacrylonitrile-based carbon fiber precursors. The reinforced materials are shown in Figure 2. The technical parameters of reinforced materials are shown in Table 4.

**Figure 3:**
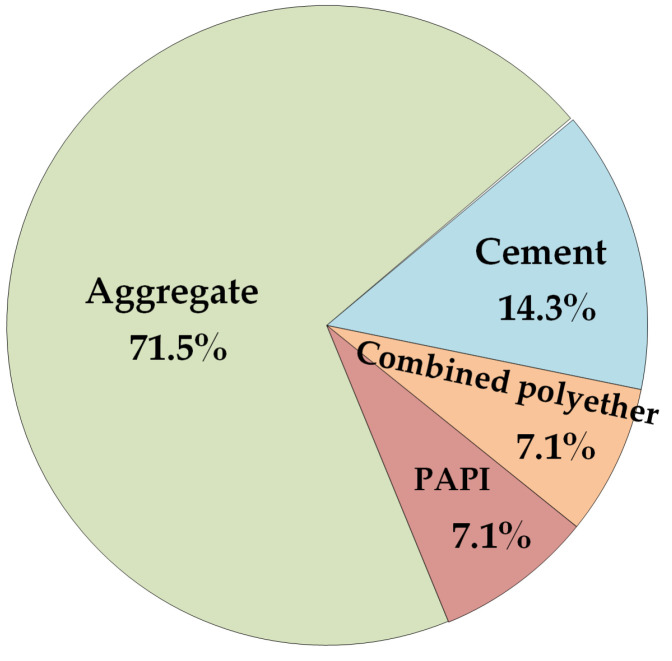
Mix proportion of polyurethane concrete (the proportion of quality in total quality).

The mix proportion of polyurethane concrete is shown in Figure 3. According to the corresponding test method in “Testing Methods of Cement and Concrete for Highway Engineering” (JTG 3420-2020) [24], the mechanical and physical properties of polyurethane concrete with this mix proportion were measured; these are presented in Table 5.

#### 2.1.2. Design Ideas

**Figure 4:**
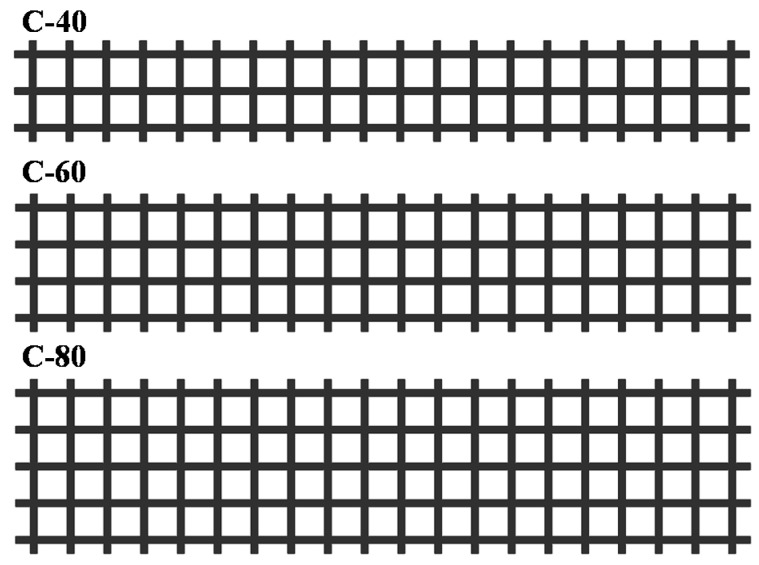
Schematic diagram of carbon fiber grid width.

**Figure 5:**
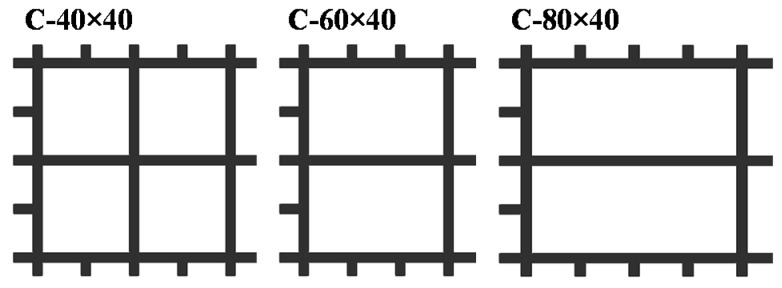
Schematic diagram of carbon fiber grid density.

To explore the influence of the type, width, number of layers, and grid density of reinforced materials on the flexural performance of polyurethane concrete, 13 groups of specimens were designed. The form of the reinforced material is shown in Figure 4 and Figure 5. The properties of the specimens are shown in Table 6.

#### 2.1.3. Preparation of Bending Specimens of Polyurethane Concrete Beams

**Figure 6:**
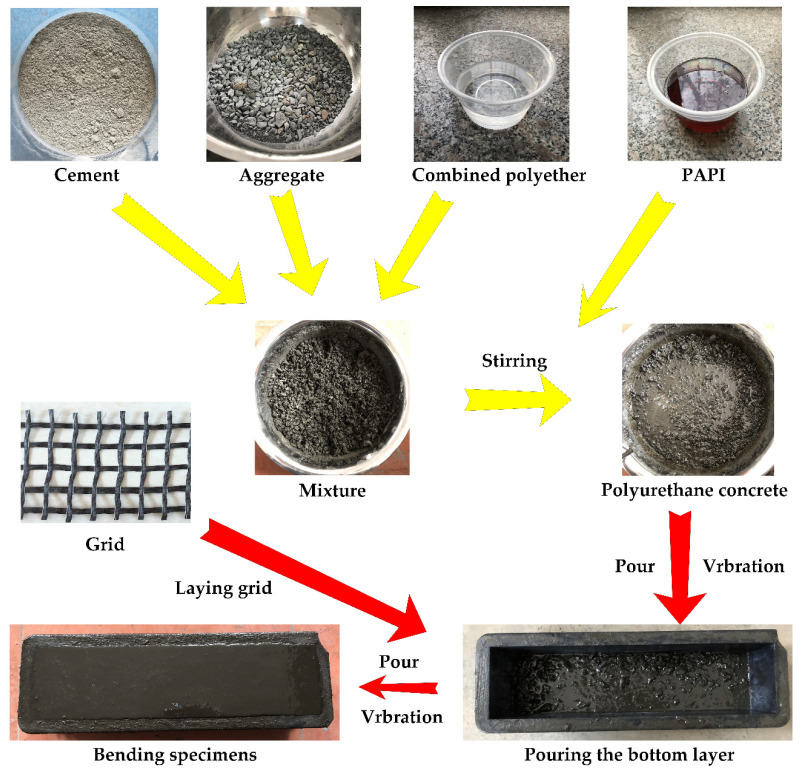
Fabrication process of the bending test piece.

(1) Fabric cutting: Cut the carbon fiber grid, steel-strand grid, and steel-wire grid required for the experiment into suitable sizes.
(2) Reinforced material handling: Fix both ends of the carbon fiber grid, steel-strand grid, and steel-wire grid on the iron fixed table. Prepare polyurethane solution, and the ratio of PAPI and combined polyether of polyurethane is 1:1. The polyurethane solution is fully mixed and evenly coated on the surface of the reinforced fabric by using a stirring rod. After the reinforced fabric is left standing for 5 min, prepare the bending specimens.
(3) Polyurethane concrete configuration: First, dry the cement and aggregate at 100~110 °C for 2 h (101-2a electrothermal blast drying oven) to completely remove the free water. Then, mix the dried aggregate and cement thoroughly with the combined polyether. Lastly, pour the PAPI into the well-mixed mixture, stir for 6 min, and prepare to pour specimens. During mixing, the laboratory temperature is 20 °C.
(4) Specimens pouring: Fix the 100 × 100 × 400 mm mold on the vibrating equipment and pour it in batches. Pour the mixed polyurethane concrete and use a steel ruler to calibrate the thickness of the pouring. After full and uniform vibration, a layer of treated reinforced material is laid on the surface. Then, pour the remaining polyurethane concrete into the mold, cover the surface of the fabric, and continue vibration. After the vibration is completed, the mold is removed from the vibration equipment, and the test is performed after curing for 24 h. The configuration process is shown in Figure 6.

**Figure 7:**
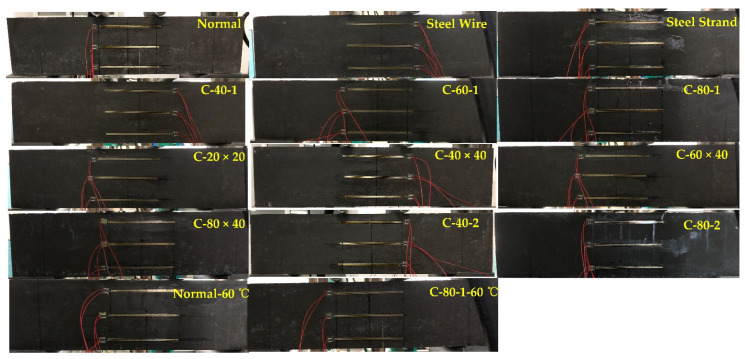
Prepared specimens.

Some of the prepared specimens are shown in Figure 7.

### 2.2. Testing Method

**Figure 8:**
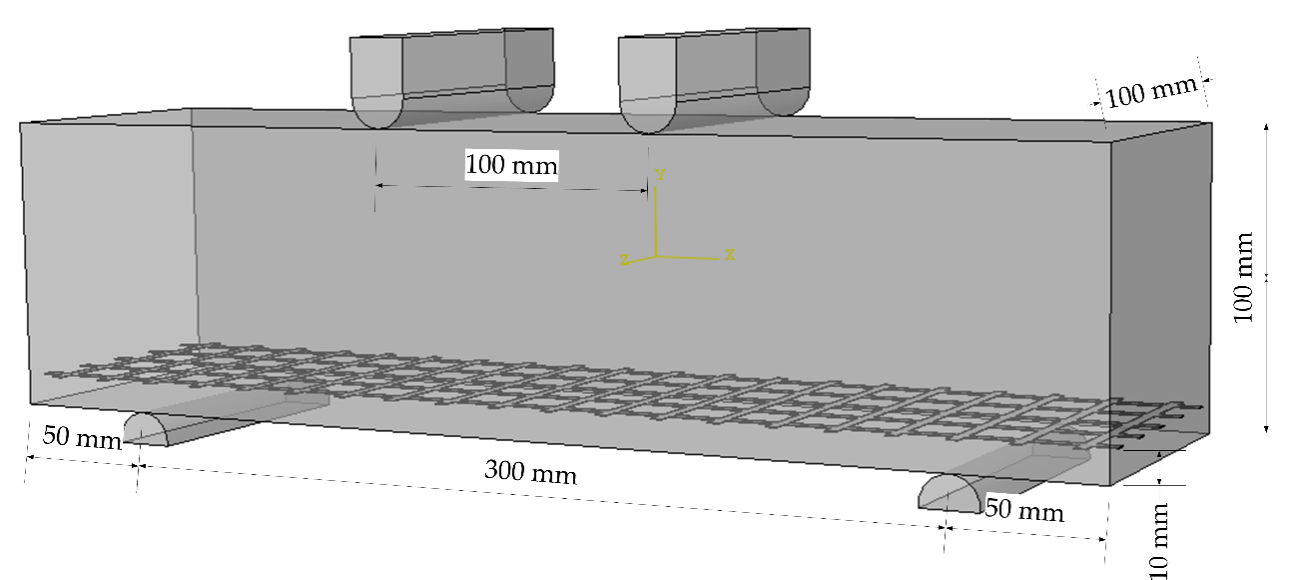
Schematic diagram of the flexural tensile test.

**Figure 9:**
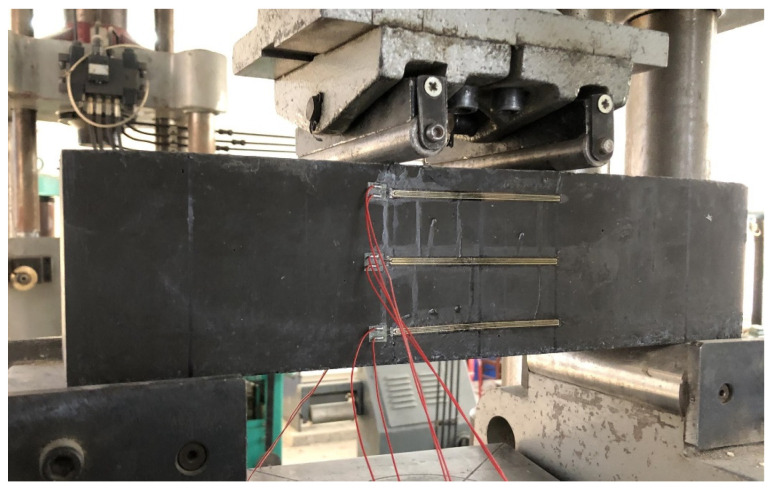
Physical map of the flexural tensile test.

The schematic diagram and physical diagram of the sample model are shown in Figure 8 and Figure 9, respectively.

**Figure 10:**
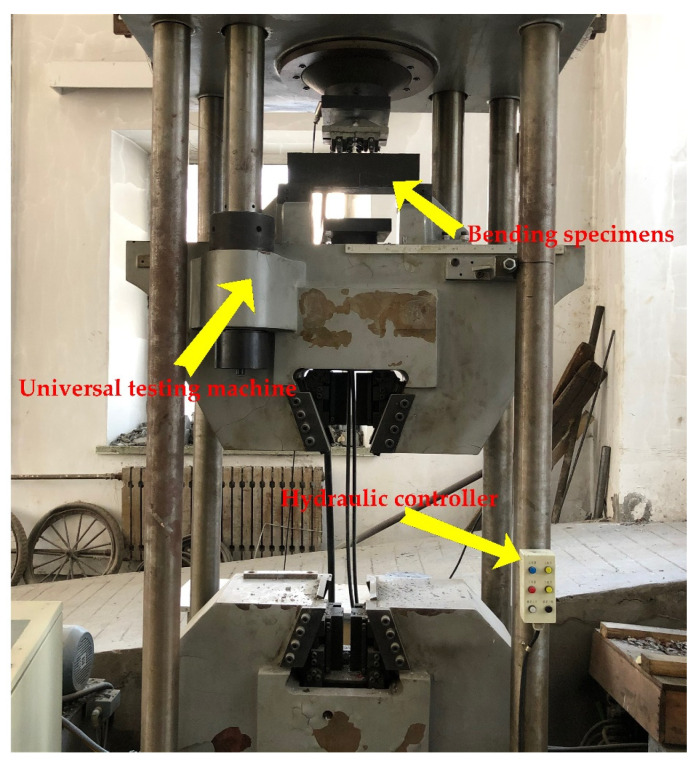
Universal testing machine.

**Figure 11:**
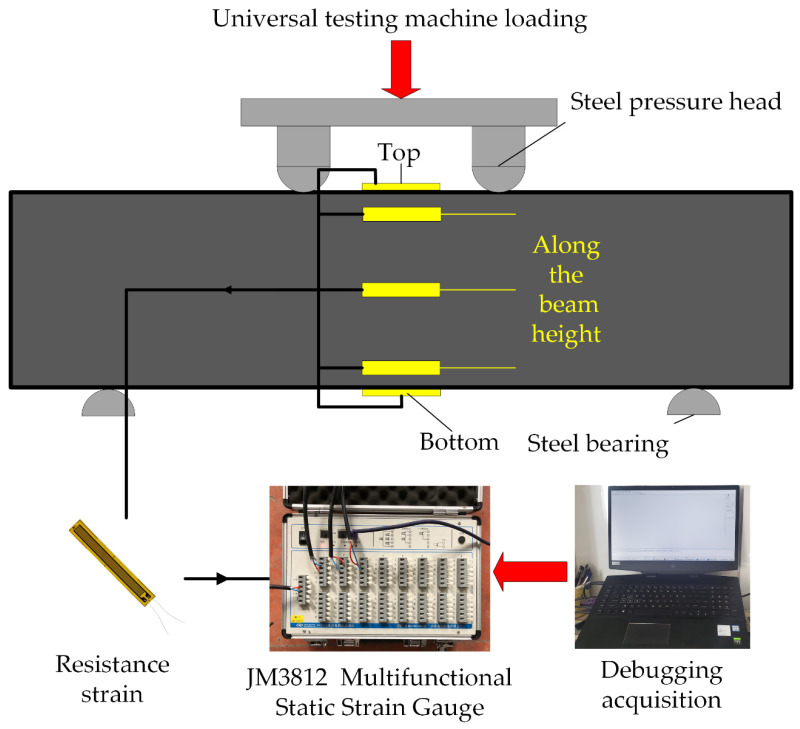
Strain acquisition system.

There are no relevant test procedures for polyurethane concrete, therefore, the four-point bending test was used to evaluate the bending performance of concrete [25,26,27,28,29]. The bending test of polyurethane concrete beam was carried out according to the T0558-2005 method in the “Testing Methods of Cement and Concrete for Highway Engineering” (JTG 3420-2020) [24], where the loading speed is 0.75 kN/s (0.075 MPa/s). First, the width and height in the middle of the specimens were measured with an accuracy of 1 mm. Then, the two movable brackets were adjusted and the specimens were placed on the bracket. After geometric alignment, the contact surface between support and the pressure bearing surface and the pressure head should be stable and uniform. The loading should be uniform and continuous. Finally, when the specimen approaches failure and begins to deform rapidly, the throttle of the testing machine shall not be adjusted until the specimen breaks, and data such as the failure load and strain shall be recorded. Testing equipment is shown in Figure 10. The strain acquisition system is shown in Figure 11. The formula for flexural strength is:

(1) $$f_{L}=\frac{FL}{bh^{2}}$$

*F*—Ultimate load (N);

*L*—Distance between supports (mm);

*b*—Specimen width (mm);

*h*—Specimen height (mm).

## 3. Results and Discussion

### 3.1. Destruction Form of the Bonding Interface

**Figure 12:**
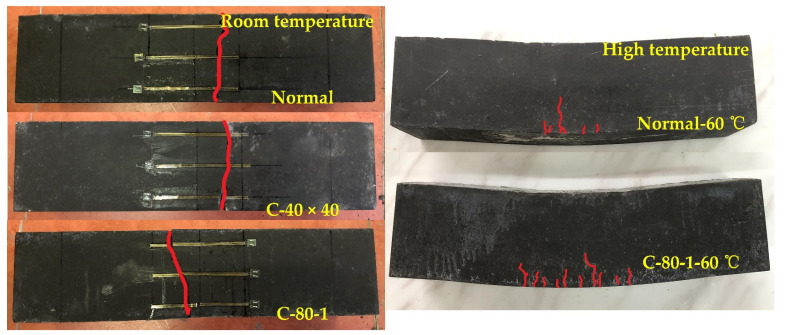
Failure patterns of specimens.

At room temperature, when the polyurethane concrete beam is subjected to load, the load–deflection curve presents a linear elastic change, which is approximately a straight line. As the increase of load, cracks appear at the bottom, and then extend vertically, and the specimen is destroyed. At high temperature, when the specimen bears the load, the load–deflection curve shows a linear elastic change before reaching the failure load. With the increase of load, small cracks appear in the middle of the bottom of the pure bending section of the specimen, but then the crack in the middle of the bottom of the Normal-60 °C specimen expands vertically upwards, and the specimen is destroyed. However, after cracks appear at the bottom of the C-80-1-60 °C specimen, with the increase of load, the crack propagation direction changes from vertical propagation to vertical and horizontal propagation. The transverse cracks propagation is accompanied by energy consumption. At the same time, the carbon fiber grid and the upper polyurethane concrete are stressed together, which improves the flexural performance of the specimens, as shown in Figure 12.

From the perspective of molecular structure, polyurethane is a kind of block polymer. Its molecular chain generally consists of two parts. At room temperature, one part is in a high elastic state, which is called soft segment. The other part is in a glassy or crystalline state, called a hard segment. Generally, the soft segment consists of flexible long chains of polymer polyol, and the hard segment consists of isocyanate. The soft segment and hard segment are alternately arranged to form a repeated structural unit. In addition to the carbamate groups, there are also a large number of polar groups in the polyurethane molecular chain, such as esters, ethers, and urea [30]. Because of the existence of these polar groups, hydrogen bond networks are formed within and among the polyurethane molecules. Therefore, polyurethane concrete presents brittle failure at room temperature. When the temperature rises, the chain segment moves thermally, the distance between the chain segment and the molecule increases, and the hydrogen bond decreases. Therefore, at high temperatures, the failure form shows plastic failure.

### 3.2. Analysis of Mechanical Properties of Experiment Material

#### 3.2.1. The Effect of Reinforced Materials on the Flexural Performance

**Figure 13:**
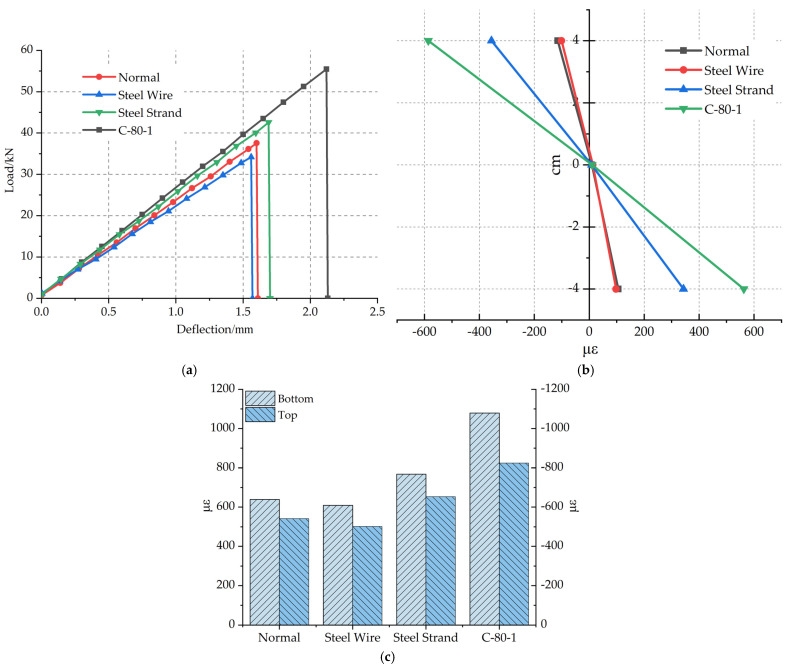
(**a**) Load–deflection curves of different reinforced materials. (**b**) Failure strain of different reinforced materials along the beam height. (**c**) Failure strain of beam bottom and top of different reinforced materials.

The load–deflection curves of polyurethane concrete specimens with different reinforced materials are shown in Figure 13a. The load–deflection curve presents a linear elastic change, which is approximate to a straight line. The average ultimate load and flexural strength of Steel Wire specimen is lower than that of the Normal specimen, and the ultimate load decreased by 9.04%. Because of the small specific surface area of the steel-wire grid material and the insufficient bonding strength of the bonding interface with polyurethane concrete, there is no synergistic effect between steel wire and polyurethane concrete. At the same time, the cross-sectional area of the specimen decreases, so the ultimate failure load is reduced. The results are consistent with the literature [31]. Compared with the Normal specimen, the average ultimate load of the Steel Strand specimen increased by 13.3% and the deflection increased by 5%. Due to the larger specific surface area of the steel-strand grid [32] and the treatment with polyurethane solution, the bonding strength is greater and the ultimate load is increased. The average ultimate load of the C-80-1 specimen reinforced by the carbon fiber grid is 47.3% higher than that of the Normal specimen. The main reason is that the surface roughness of carbon fiber grid surface is large. It is beneficial to form an effective mechanical interlock at the interface joint and form a bidirectional force on a carbon fiber grid [33]. In addition, due to the uniform transfer of internal load, the flexural performance of the specimen is improved.

The failure strains of specimens with different reinforced materials along beam heights are shown in Figure 13b. At 4 cm below the neutral axis, the failure strain of the Steel Wire specimen decreased by 7.5% compared with that of the Normal specimen, that of the Steel Strand specimen increased by 223.6% compared with the Normal specimen, and that of the C-80-1 specimen increased by 431.1% compared with the Normal specimen.

The average failure strains at the bottom of the beam are shown in Figure 13c. The Steel Wire specimen is 4.7% lower than that of the Normal specimen. The average failure strain at the bottom of the Steel Strand specimen is slightly higher than that of the Normal specimen. The average failure strain at the bottom of the C-80-1 specimen is 68.9% higher than that of the Normal specimen. Compared with steel-wire grid and steel-strand grid, carbon fiber grid has a higher effect on improving the flexural performance of polyurethane concrete. 

#### 3.2.2. The Effect of Reinforced Material Width on the Flexural Performance

**Figure 14:**
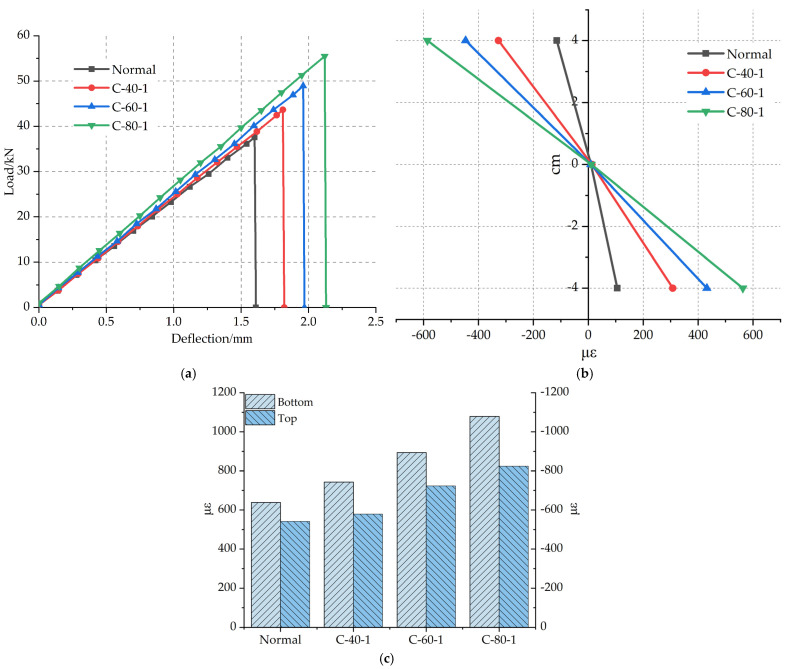
(**a**) Load–deflection curves of different grid widths. (**b**) Failure strain along the beam height for different grid widths. (**c**) Failure strain at the bottom and top of beams with different grid widths.

The load–deflection curves of specimens with reinforced materials of different grid widths are shown in Figure 14a. Figure 4 shows the grids with different widths. The average ultimate load of the C-40-1 specimen increased by 10.6% compared with the Normal specimen, that of the C-60-1 specimen increased by 16.2% compared with the C-40-1, and that of the C-80-1 specimen is higher than the C-60-1 specimen increased by 13.4%. The average ultimate load of C-80-1 increased by 47.7% than that of the Normal specimen. The reason is that the wider the grid, the more longitudinal carbon fiber strips, which has an excellent synergy with polyurethane concrete to bear a load, thereby improving the flexural performance of the specimen, such as that studied by Zena Aljazaeri et al. [31]. In addition, the transverse carbon fiber strips also enable the transverse force to be transmitted better when being stressed.

The failure strains along beam height of specimens with different carbon fiber grid widths are shown in Figure 14b. At 4 cm below the neutral axis, the failure strain of the C-40-1 specimen increased by 190.6% than that of the Normal specimen, that of the C-60-1 specimen increased by 40.3% compared with the C-40-1 specimen, and that of the C-80-1 specimen increased by 30.3% compared with the C-60-1 specimen.

The average failure strains at the bottom of the beam are shown in Figure 14c. The failure strain increases with the increase of the width of carbon fiber grid, and the average failure strain at the bottom of the C-80-1 specimen increased by 64.2% compared with the Normal specimen. The width of the carbon fiber grid is one of the important factors affecting the flexural performance of polyurethane concrete.

#### 3.2.3. The Influence of Numbers of Layers of Reinforced Material on the Flexural Performance

**Figure 15:**
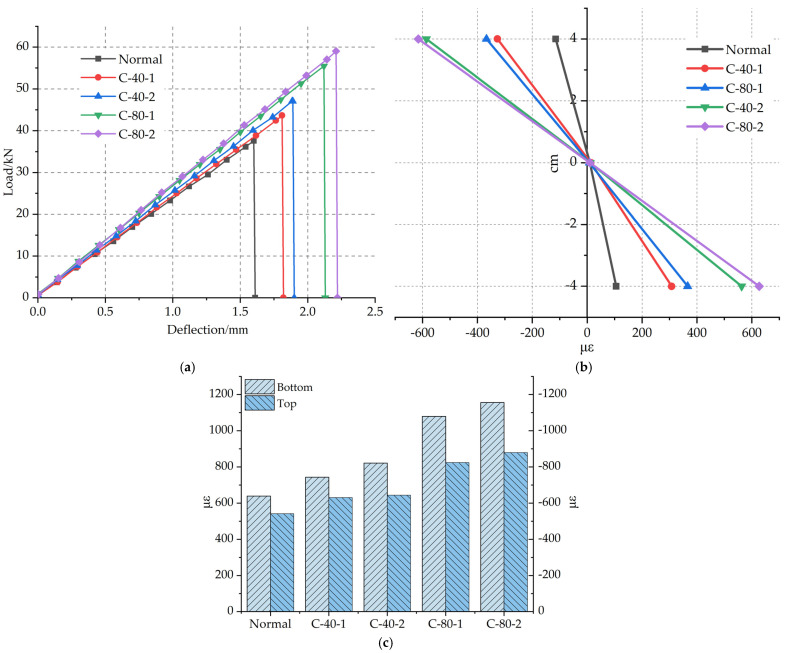
(**a**). Load–deflection curve for the number of different reinforced layers. (**b**). Failure strain along the beam height for the number of different reinforced layers. (**c**). Failure strain at the bottom and top of beams for the number of different reinforced layers.

Figure 15a–c shows the influence of the number of reinforcement layers on the flexural performance of polyurethane concrete beam. It shows the flexural capacity of single-layer and double-layer reinforcement layers under C-40 and C-80 conditions. The load–deflection curves of specimens with different numbers of reinforcement layers are shown in Figure 15a. From a statistical point of view, when the width is 40 mm, the average ultimate load of the C-40-2 specimen increased by 7.8% than that of the C-40-1 specimen. When the width is 80 mm, the average ultimate load of C-80-2 specimen increased by 6.4% than that of C-80-1 specimen. The results show that the number of layers of carbon fiber grid will affect the flexural bearing capacity of polyurethane concrete. Sukpyo Kang et al. [34] tested the effect of different layers of grids on the flexural bearing capacity of concrete. The test results are in line with the conclusions obtained by Suppyo Kang. However, compared with the single-layer grid, the lifting effect of the double-layer grid did not increase proportionally as expected. Because the upper layer is close to the neutral axis, the tensile force on the upper grid is less than that on the lower grid when the specimen is bent, so the reinforcement effect of the double-layer carbon fiber grid is 1.5 times smaller than that of the single-layer carbon fiber gird. Therefore, the optimum reinforcement layer of the carbon fiber grid is one layer.

The average failure strains at the bottom of the beam are shown in Figure 15c. The average failure strain at the bottom of the C-40-2 specimen increased by 10.5% compared with that of the C-40-1 specimen. The average strain at the bottom of the C-80-2 specimen increased by 7% compared with that of the C-80-1 specimen.

#### 3.2.4. Influence of the Grid Density of the Reinforced Material on the Flexural Performance

**Figure 16:**
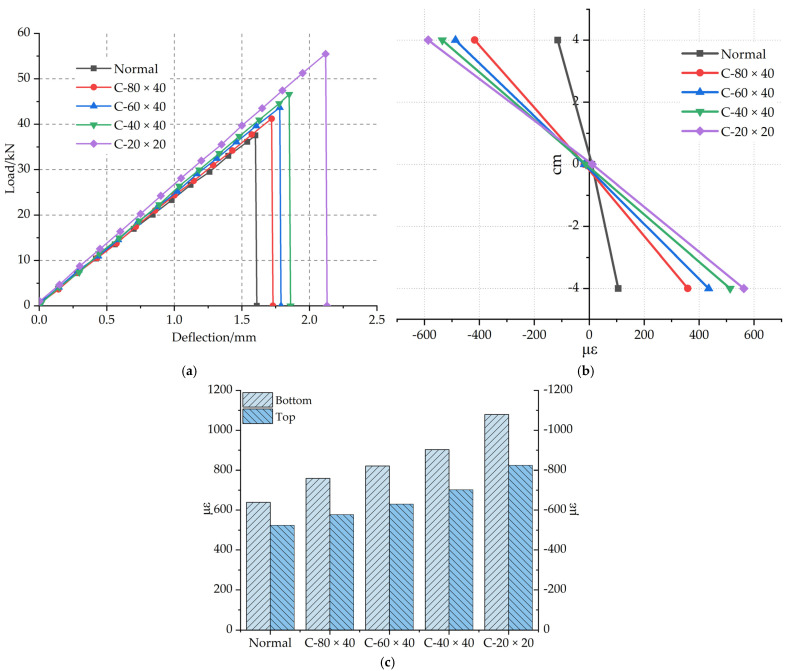
(**a**) Load–deflection curves of different grid densities. (**b**) High failure strain along the beam height with different grid densities. (**c**) Failure strain at the bottom and top of beams with different grid densities.

The grid density of carbon fiber grid also affects the flexural performance of polyurethane concrete [35], as shown in Figure 16a–c. The carbon fiber grids with different grid densities are shown in Figure 5. The load–deflection curves of specimens with the reinforcement of different carbon fiber grid’s grids density are shown in Figure 16a. The average ultimate load of the C-20 × 20 specimen increased by 47.7% compared with the Normal specimen. The average ultimate load of the C-40 × 40 specimen increased by 24% compared with the Normal specimen. The main reason for the difference in enhancement performance between the two grid densities is the difference in longitudinal strips. The number of longitudinal strips of C-60 × 40 specimens is the same as that of C-40 × 40 specimens. The main difference is that the number of horizontal strips is different, which leads to different transverse force transmission. Compared with the Normal specimen, the average ultimate load of C-60 × 40 increased by 16.2%, and that of the C-80 × 40 specimen increased by 9.7%. This indicates that the grid density will impact flexural performance of polyurethane concrete. The higher the density of the carbon fiber grid, the better the improvement effect on the flexural performance of polyurethane concrete.

The average failure strain at the bottom of the beam is shown in Figure 16c. Compared with that of the Normal specimen, the average failure strain at the bottom of the C-20 × 20 specimen increased by 68.9%, that of the C-40 × 40 specimen increased by 41.3%, that of the C-60 × 40 specimen increased by 28.5%, and that of the C-80 × 40 specimen increased by 18.8%. The optimal grid density to improve the flexural performance of polyurethane concrete is 20 × 20 mm.

#### 3.2.5. Ductility Test

Ductility is also one of the important factors for evaluating the deformability of specimens. The study of related RC beams uses ductility to evaluate the bending performance, such as Karimipour Arash et al. [27,28]. So the ductility ratio (DR) index is adopted to measure the ductility of polyurethane concrete.

(2) $$i=\frac{\Delta_{0.85}}{\Delta y}$$

**Figure 17:**
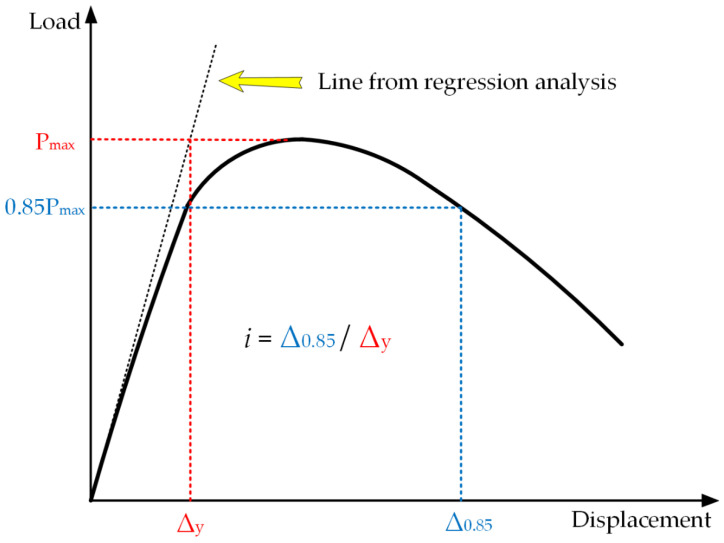
Definition of the displacement–ductility ratio [29].

**Figure 18:**
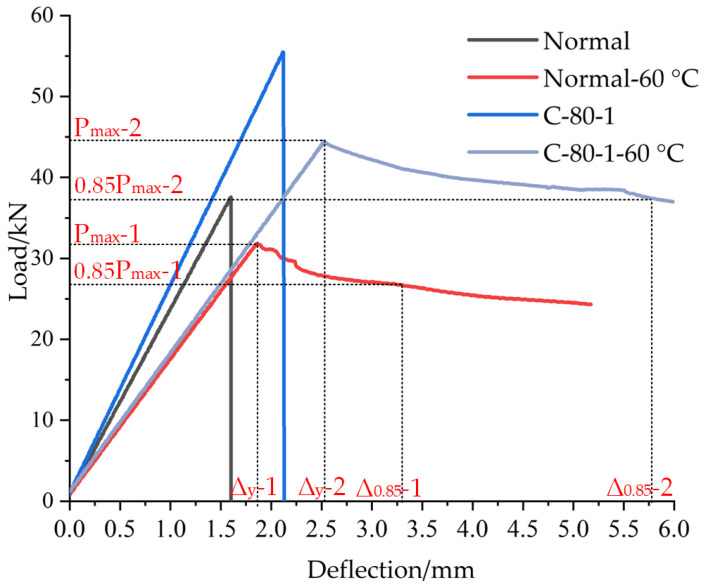
Load–deflection curves of specimens.

Cohn and Bartlett [36] defined it as the ratio between the displacement corresponding to 85% of the maximum bending capacity in the post-peak portion of the curve and the one corresponding to the first yield displacement of a beam, as shown in Figure 17 and Equation (2) [26]. When the temperature of the specimen was 60 °C, the influence of carbon fiber grid on the flexural bearing capacity and ductility of polyurethane concrete was tested. The load–deflection curves of specimens are shown in Figure 18.

Under the conditions of high-temperature, the failure form of polyurethane concrete is plastic. The effect of carbon fiber grid on the flexural performance and ductility of polyurethane concrete was tested at 60 °C. The ultimate load of the C-80-1-60 °C specimen is 19.7% lower than that of C-80-1 specimen and still 40.1% higher than that of Normal-60 °C specimen, and i-2 is 35.7% higher than i-1, as shown in the Figure 18. Therefore, under high-temperature conditions, the ultimate load of the specimen will be reduced, but the carbon fiber grid will still improve the flexural performance of the polyurethane concrete, while showing better ductility.

## 4. Conclusions

Through the four-point bending test, the polyurethane concrete beams were tested, in order to obtain how the carbon fiber grid can improve flexural bearing capacity of polyurethane concrete. By analyzing the test results, the following conclusions can be drawn:

(1) The reinforcement of carbon fiber grid on the flexural performance of polyurethane concrete is better than that of the steel-wire grid and steel-strand grid. The flexural strength of polyurethane concrete was reduced by laying of steel-wire grid. Compared with the Normal specimen, the flexural strength of the specimens reinforced by the carbon fiber grid and steel-strand grid increased by 47.7 and 13.3%, respectively.
(2) The average ultimate load of the polyurethane concrete beam reinforced by the carbon fiber grid increased by 47.3% compared with that of the Normal specimen. The average failure strain of polyurethane concrete beam reinforced by the carbon fiber grid increased by 68.9% compared with that of the Normal specimen.
(3) The best reinforcement layer of carbon fiber grid for strengthening the flexural capacity of polyurethane concrete is one layer, and the best reinforcement width is 80 mm. The number of longitudinal carbon fiber strips plays a significant role.
(4) The density of the carbon fiber grid also affects the enhancement of flexural performance of polyurethane concrete. The optimal grid density is 20 × 20 mm, which shows better transverse force transmission than the other grid densities, and is more conducive to improving the flexural capacity of polyurethane concrete.

## Figures and Tables

**Table 1:** Physical and chemical properties of combined polyether.

NO.	Category	Index
1	Appearance	Colorless Transparent Liquid
2	Viscosity (25 °C) (mPa·s)	200–1500
3	Hydroxyl value (mg KOH/g)	30.5–32.0
4	Density (25 °C) (g/cm^3^)	1.11 ± 0.20

**Table 2:** Technical index of AC-10 fine-grained concrete.

Mesh Size (mm)	9.5	4.75	2.36	1.18	0.6	0.3	0.15	0.075	Mineral Powder
Percentage Through Sieve	95%	60%	44.0%	32.0%	22.5%	16.0%	11.0%	6.0%	—
Aggregate Mass (per 1000 g)	50.0	380.0	319.2	170.5	62.2	15.2	2.6	0.3	0.02

**Table 3:** Physical and mechanical properties of cement.

Flexural Strength		Compressive Strength		Setting Time		Fineness
3 Days	28 Days	3 Days	28 Days	Initial Setting Time	Final Setting Time	
4.6 MPa	10.5 MPa	24.6 MPa	55.6 MPa	240 min	276 min	1.8%

**Table 4:** Technical parameters of reinforced materials.

Reinforced Materials	Ultimate Breaking Load	Design Tensile Strength	Elastic Modulus	Theoretical Cross-Sectional Area
Carbon fiber Grid	3200 N	3600 MPa	240 kN·mm^−2^	44 mm^2^
Steel-Strand Grid	1800 N	1596 MPa	139 kN·mm^−2^	39.45 mm^2^
Steel-Wire Grid	680 N	342 MPa	180 kN·mm^−2^	20.31 mm^2^

**Table 5:** Mechanical and physical properties of polyurethane concrete.

	Average	Standard Deviation	Coefficient of Variation (%)
Density (kg/m^3^)	1762.2	15.3	0.866
Compression Strength (MPa)	59.9	1.0	1.743
Tensile Strength (MPa)	42.0	0.8	1.961

**Table 6:** Properties of the specimens.

Specimen Number	Reinforced Material	Number of Reinforcement Layers	Grid Width	Grid Density	Temperature
Normal	None	\	\	\	20 °C
Normal-60 °C					60 °C
Steel Wire	Steel-Wire Grid	1	80 mm	\	20 °C
Steel Strand	Steel-Strand Grid	1	80 mm	\	
C-40-1	Carbon fiber Grid	1	40 mm	20 × 20 mm	
C-60-1			60 mm		
C-80-1/C-20 × 20			80 mm		20 °C
C-80-1-60 °C					60 °C
C-40 × 40		1		40 × 40 mm	20 °C
C-60 × 40				60 × 40 mm	
C-80 × 40				80 × 40 mm	
C-40-2		2	40 mm	20 × 20 mm	
C-80-2			80 mm		

## Data Availability

Not applicable.
